# Supplementary material for: OCIAD2 as a novel prognostic and therapeutic biomarker for pancreatic cancer: A study based on transcriptomic signature and bioinformatics analysis
Source: PLoS Comput Biol. 2025 Oct 7;21(10):e1013566. doi: 10.1371/journal.pcbi.1013566 (PMC12517509; doi:10.1371/journal.pcbi.1013566)
Supplement: S1 Text — Information of 7 GEO datasets for differential expression gene analysis. Table B. Significantly up-regulated and down-regulated DEGs in each GEO dataset. (DOCX) [file pcbi.1013566.s001.docx]

**Table A in S1 Text. Information of 7 GEO datasets for differential expression gene analysis.**

| **GEO datasets** | **Countries** | **Total cases** | **Tumor** | **Adjacent normal** | **Publication time** |
| --- | --- | --- | --- | --- | --- |
| GSE102238 | China | 100 | 50 | 50 | 2017 |
| GSE183795 | America | 241 | 139 | 102 | 2022 |
| GSE71729 | America | 191 | 145 | 46 | 2015 |
| GSE62452 | America | 130 | 69 | 61 | 2014 |
| GSE28735 | America | 90 | 45 | 45 | 2012 |
| GSE62165 | Belgium | 131 | 118 | 13 | 2016 |
| GSE60980 | Norway | 61 | 49 | 12 | 2014 |

**Table B in S1 Text. Significantly up-regulated and down-regulated DEGs in each GEO dataset.**

| GEO dataset | Up-regulated | Down-regulated |
| --- | --- | --- |
| GSE102238 | 3053 | 1291 |
| GSE183795 | 619 | 271 |
| GSE71729 | 499 | 312 |
| GSE62452 | 689 | 302 |
| GSE28735 | 754 | 448 |
| GSE62165 | 2766 | 1919 |
| GSE60979 | 2263 | 1735 |
