# Supplementary material for: OCIAD2 as a novel prognostic and therapeutic biomarker for pancreatic cancer: A study based on transcriptomic signature and bioinformatics analysis
Source: PLoS Comput Biol. 2025 Oct 7;21(10):e1013566. doi: 10.1371/journal.pcbi.1013566 (PMC12517509; doi:10.1371/journal.pcbi.1013566)
Supplement: S2 Text — (DOCX) [file pcbi.1013566.s002.docx]

**The mRNA expression levels of *DCBLD2*, *OCIAD2*, and *SAMD9* in various tissues by single-cell sequencing.**

These datasets contained single-cell sequencing data of normal pancreas, adjacent normal, primary, and metastatic tumor tissues. The data were analyzed by R software (version 4.3.0, The R Foundation for Statistical Computing, Vienna, Austria). After loading the data into R, we first built the Seurat object using the CreateSeuratObject function (min.cells=10, min.features= 200) of the Seurat (version 5.0.3) package. Next, the cells were filtered for quality control using the following parameters: nFeature_RNA (number of genes expressed per cell) > 500 & nCount_RNA (number of Unique Molecular Identifiers [UMI] per cell) > 1000 & nCount_RNA < 20000 & percent.mt (fraction of UMIs aligning to mitochondrial genes) < 10. The NormalizeData function standardizes the data. The FindVariableFeatures and ScaleData functions were used for variable gene screening and normalization. The RunPCA function was used for PCA dimension reduction, and the RunHarmony function of the harmony (version 1.2.0) package was used to correct the batch effect of different data sets. The FindNeighbors (dims = 1:30) and FindClusters (resolution = 0.4) functions were used for cell clustering and grouping. The RunTSNE function was used for dimensionality reduction visualization. Finally, the FindMarkers function was used to analyze marker genes. Cell type definition was identified by the following marker genes: T cells (*IL7R*, *CD3D*, *CD3E*, *CD2*), ductal cells (*KRT19*, *EPCAM*, *KRT7*, *ANXA4*, *SLC4A4*, *CFTR*), macrophages (*AIF1*, *CD68*, *CD14*), acinar cells (*PRSS1*, *REG1B*, *CPA1*, *CTRB2*, *CTRB1*, *CLPS*), MDSCS (*S100A8*, *S100A9*, *S100A12*), fibroblasts (*DCN*, *LUM*, *BGN*, *SPARC*, *COL1A1*), B cells (*CD79A*, *MS4A1*, *MZB1*), endothelial cells (*PLVAP*, *PECAM1*), mast cells (*CPA3*, *KIT*), stellate cells (*ACTA2*, *ADIRF*, *PDGFRB*), Schwann cells (*CDH19*, *S100B*, *CRYAB*, *PLP1*). The mRNA expression of *DCBLD2*, *OCIAD2,* and *SAMD9* was further analyzed and visualized by Dittoseq (Version 1.14.2) and Scientomize (Version 2.1.2) package.
