# Supplementary figures and images for: OCIAD2 as a novel prognostic and therapeutic biomarker for pancreatic cancer: A study based on transcriptomic signature and bioinformatics analysis
Source: PLoS Comput Biol. 2025 Oct 7;21(10):e1013566. doi: 10.1371/journal.pcbi.1013566 (PMC12517509; doi:10.1371/journal.pcbi.1013566)

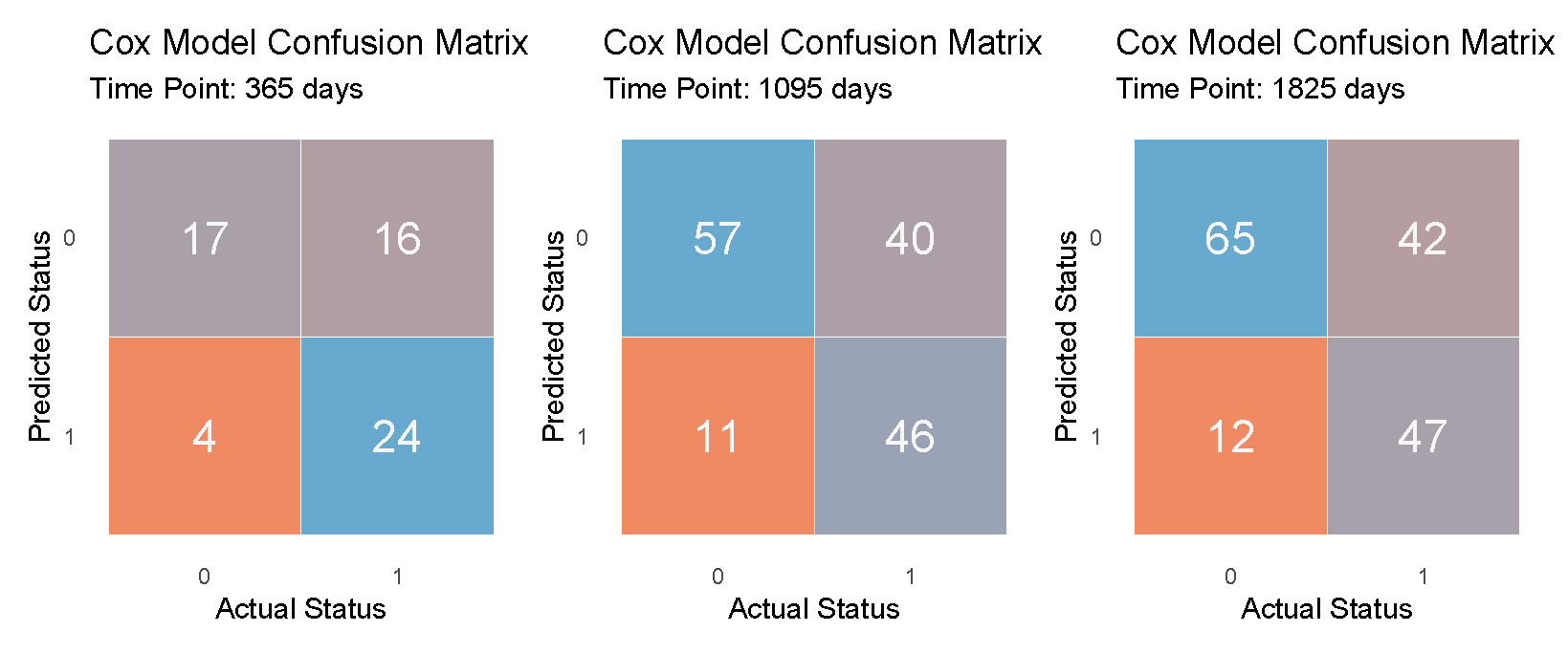

Supplement: S1 Fig — (TIF) [file pcbi.1013566.s004.tif]

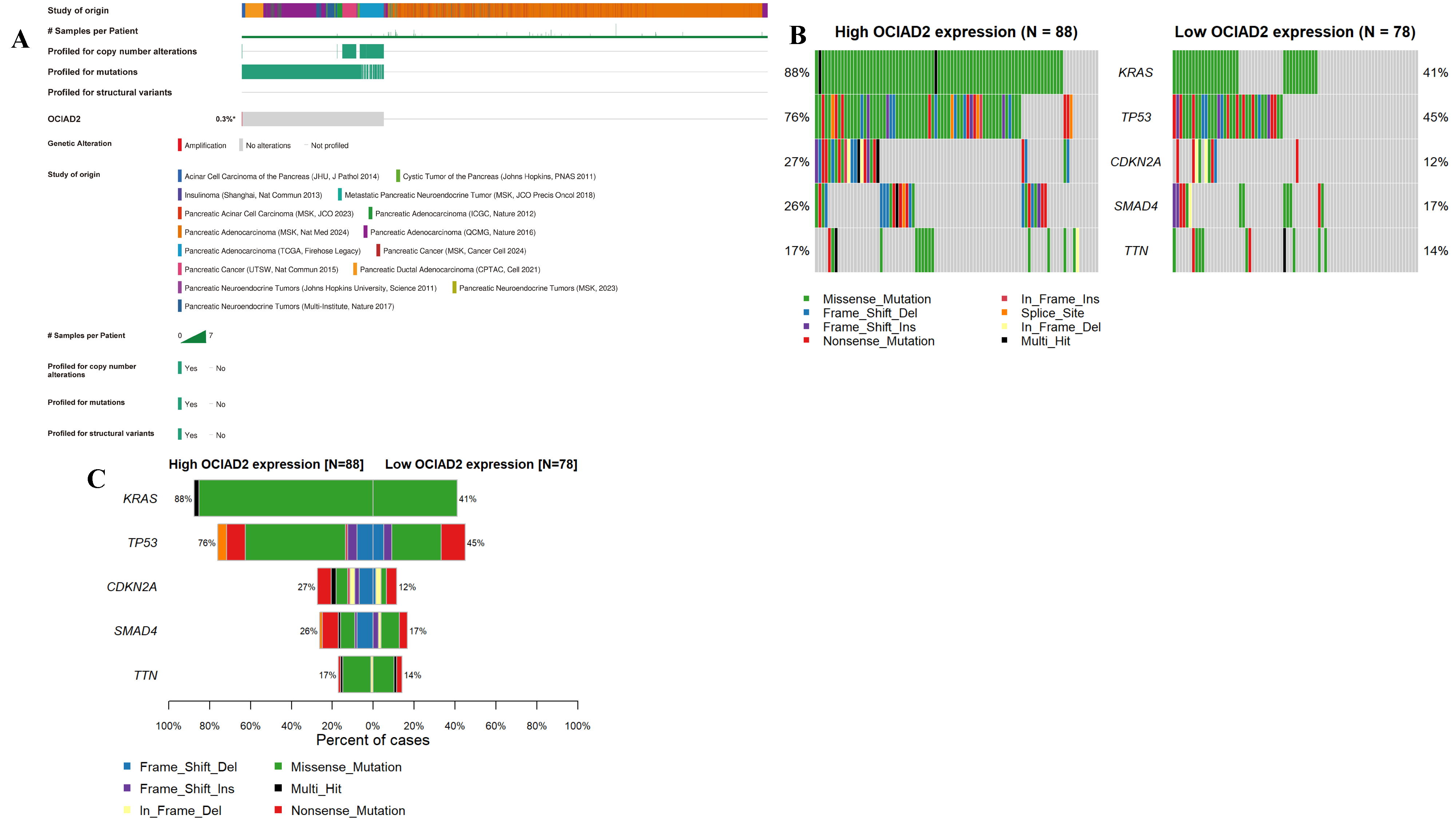

Supplement: S2 Fig — (B-C) Mutation profiles of PC patients with different OCIAD2 expression levels. (TIF) [file pcbi.1013566.s006.tif]
