# Supplementary material for: OCIAD2 as a novel prognostic and therapeutic biomarker for pancreatic cancer: A study based on transcriptomic signature and bioinformatics analysis
Source: PLoS Comput Biol. 2025 Oct 7;21(10):e1013566. doi: 10.1371/journal.pcbi.1013566 (PMC12517509; doi:10.1371/journal.pcbi.1013566)
Supplement: Attachment — Submitted filename: Response to Reviewers.docx [file pcbi.1013566.s009.docx]

Dear editor，

Thanks to the editor and reviewers' expert comments, which are of great assistance in improving the quality of the manuscript. We carefully revised the manuscript and further clarified the scientific logic within the writing. We respond to the reviewers' comments point by point and use tracked changes in the revised manuscript.

**Reviewers' comments:**

**Reviewer 1**

In this article, extensive research was conducted on pancreatic cancer through a large amount of transcriptome data. The results revealed many genes that are poorly understood but potentially important in pancreatic cancer. Further studies in the BxPC3 cell line based on high-throughput sequencing revealed a relationship between OCIAD2 and the over-activation of STAT and cell cycle pathways. Furthermore, its conclusion has been verified in the transcriptome and clinical data of pancreatic cancer in other independent studies. These findings have good clinical significance and deserve attention. However, the following issues still need to be further addressed:

1. The mutation of OCIAD2 in pancreatic cancer can be analyzed at the genomic level. For example, the mutation of OCIAD2 itself in PC, and the characteristics of the mutation spectrum in patients with different OCIAD2 expression levels.

Response: Thank you for reviewing this manuscript, and the following is our supplementary analysis. Based on the cBioPortal database, we analyzed 15 studies comprising a total of 4,102 samples from 4,045 patients. It was found that only 0.3% of the patients developed genetic alterations (Supplement 5). Then, using the TCGA pancreatic cancer data, we divided *OCIAD2* into high- and low-expression groups and explored the mutation differences between the two groups. It was found that the proportion of gene mutations such as KRAS, TP53, and *CDKN2A* was higher in the patients in the high *OCIAD2* expression group (Supplement 5). We have supplemented this in the discussion section.

Lines 426-429: “In addition, we found that *OCIAD2* itself was rarely mutated, but PC patients with high *OCIAD2* expression had more mutations in *KRAS*, *TP53*, and *CDKN2A* than those with low *OCIAD2* expression (Supplement 5)”.

2. Gene set enrichment analysis should be abbreviated as GSEA when it first appears in the method。

Response: In the resubmitted manuscript, we have corrected this deficiency.

Lines 153-155: “The clusterProfiler (version 4.10.1) package (23) was used for GO, KEGG, and Gene Set Enrichment Analysis (GSEA)”.

3. Figure 5J shows incomplete and needs to be resized.

Response: Revisions have been made in the resubmitted manuscript.

Figure 5. The mechanism of targeting *OCIAD2* to inhibit the progression of pancreatic cancer. (A) The expression level of *OCIAD2* mRNA in the siRNA interference group in the BxPC-3 cell line was significantly down-regulated compared with the control group. (B) Principal component analysis results at the transcriptome level of the two groups of samples. (C) The activation of 14 signaling pathways in the *OCIAD2* knockdown group. (D-E) Volcano plot and heat map of up-and down-regulated DEGs in the *OCIAD2* knockdown group. (F) The activities of JAK-STAT, PI3K, NFkB, and Androgen pathways in the *OCIAD2* knockdown group. (G) KEGG enrichment analysis of down-regulated DEGs in the OCIAD2 knockdown group. (K) DEGs enriched in each major pathway in KEGG analysis. (I-J) GSEA analysis results of DEGs after *OCIAD2* knockdown.

4. Some expressions in the method are not standardized. For example, the writing of "siOCIAD2" in the method RNA sequencing and bioinformatics analysis is unclear and needs to be corrected.

Response: Revisions have been made in the resubmitted manuscript.

Lines 143-146: “To explore the potential mechanism *OCIAD2* promotes progress in PC, we conducted the transcriptome sequencing and analysis. siRNA targeting knockdown of *OCIAD2* and negative controls were transfected into BxPC-3 cells. After 48 hours, cells from each group were collected, and TRIzol reagent was added to extract total RNA”.

5. Given the complex role of OCIAD2 in tumors such as lung cancer, the discussion of the relationship between OCIAD2 and clinicopathological features of PC patients should be increased.

Response: In the discussion section, we have added content on the clinical characteristics of patients with *OCIAD2* and PC.

Lines 424-429: “In this study, we found that the expression level of OCIAD2 was strongly correlated with chronic pancreatitis, primary therapy outcome, and pathological type in PC patients. In addition, we found that OCIAD2 itself was rarely mutated, but PC patients with high OCIAD2 expression had more mutations in KRAS, TP53, and CDKN2A than those with low OCIAD2 expression (Supplement 5)”.

6. It is suggested to further refine the language of the article.

Response: In this revised manuscript, we have again carefully checked and revised the language. For example, in the introduction section, Lines 64-66: “Several currently reported biomarkers stem from small, heterogeneous tumor samples, without extensive validation, posing a challenge to their reliability”. In the Method section, Lines 79-81: “We used the keywords “pancreatic cancer”, “pancreatic ductal adenocarcinoma”, “pancreatic ductal carcinoma”, or “Pancreatic adenocarcinoma (PAAD)” to search the Gene Expression Omnibus (GEO) database”. In the results section, Lines 224-225: “We constructed a Nomogram to predict the 1,3, and 5-year survival rates of patients based on the Cox model”. More revisions are shown in "Revised Manuscript with Track Changes".

**Reviewer 2**

The study of "OCIAD2 as a novel prognostic and therapeutic biomarker for pancreatic cancer: a study based on transcriptomic signature and bioinformatics analysis" combined multiple datasets of pancreatic cancer and identified OCIAD2 as a poential biomarker for prognosis and therapy. However, the value of this study is still limited.

1. There have already been too many similar reports for pancreatic cancer, which severely challenged the novelty of this study. Just name a few (still many others):

1) JagadeeswaraRao 2024 An integrated ensemble learning technique for gene expression classification and biomarker identification from RNA-seq data for pancreatic cancer prognosis, International Journal of Information Technology.

2) Hossen 2023 Robust identification of common genomic biomarkers from multiple gene expression profiles for the prognosis, diagnosis, and therapies of pancreatic cancer, Computers in Biology and Medicine.

3) Zhuang 2021 Identification of LIPH as an unfavorable biomarkers correlated with immune suppression or evasion in pancreatic cancer based on RNA-seq, Cancer Immunology, Immunotherapy.

4) Xu 2021 Development and clinical validation of a novel 9-gene prognostic model based on multi-omics in pancreatic adenocarcinoma, Pharmacological Research.

5) Atay 2020 Integrated transcriptome meta-analysis of pancreatic ductal adenocarcinoma and matched adjacent pancreatic tissues, PeerJ.

6) Zhang 2020 Early Diagnosis of Pancreatic Ductal Adenocarcinoma by Combining Relative Expression Orderings With Machine-Learning Method, Frontiers in Cell and Developmental Biology.

7) Cheng 2019 Identification of candidate diagnostic and prognostic biomarkers for pancreatic carcinoma, eBioMedicine.

Response: Dear professor, thank you for reviewing our article and offering suggestions. The following is our reply. Although many studies (including the ones you mentioned above) are based on transcription, their research purposes, analytical strategies, and methods used, as well as the final results, are not entirely consistent. The following are the main results and concerns of these articles that we have summarized:

1) JagadeeswaraRao 2024 An integrated ensemble learning technique for gene expression classification and biomarker identification from RNA-seq data for pancreatic cancer prognosis, International Journal of Information Technology

This study only used the TCGA-PAAD data for differential analysis, and they focused on BUB1, BUB1B, KIF11, and TTK genes, which were different from those in our report.

2) Hossen 2023 Robust identification of common genomic biomarkers from multiple gene expression profiles for the prognosis, diagnosis, and therapies of pancreatic cancer, Computers in Biology and Medicine

In this paper, the authors used four datasets: GSE15471, GSE16515, GSE71989, and GSE22780. The analysis identified eight cDEGs (ADAM10, COL1A2, FN1, P4HB, ITGB1, ITGB5, ANXA2, and MYOF) as the key genes responsible for PC, which were different from the genes we reported.

3) Zhuang 2021 Identification of LIPH as an unfavorable biomarkers correlated with immune suppression or evasion in pancreatic cancer based on RNA-seq, Cancer Immunology, Immunotherapy

This article focuses on LIPH based on transcriptome data. It's still completely different from ours.

4) Xu 2021 Development and clinical validation of a novel 9-gene prognostic model based on multi-omics in pancreatic adenocarcinoma, Pharmacological Research

In this study, researchers report the use of nine characteristic genes, including UNC13B, TSPYL4, MICAL1, KLHDC7B, KLHL32, AIM1, ARHGAP18, DCBLD1, and CACNA2D4, for risk stratification. None of these genes were identical to those reported in our manuscript.

5) Atay 2020 Integrated transcriptome meta-analysis of pancreatic ductal adenocarcinoma and matched adjacent pancreatic tissues, PeerJ

The 21 prognostic genes identified in this study were ITGB6, LAMC2, KRT7, SERPINB5, IGF2BP3, IL1RN, MPZL2, SFTA2, MET, LAMA3, ARNTL2, SLC2A1, LAMB3, COL17A1, EPSTI1, IL1RAP, AK4, ANXA2, S100A16, KRT19 and GPRC5A were still different from those reported in our study.

6) Zhang 2020 Early Diagnosis of Pancreatic Ductal Adenocarcinoma by Combining Relative Expression Orderings With Machine-Learning Method, Frontiers in Cell and Developmental Biology

This is a study of early PDAC diagnostic biomarkers based on transcriptome, and its purpose and results are completely different from ours.

7) Cheng 2019 Identification of candidate diagnostic and prognostic biomarkers for pancreatic carcinoma, eBioMedicine

The main findings of this article are that MC7, TMPRSS4, SCEL, SLC2A1, CENPF, SERPINB5, and SLC6A14 may be potential biomarkers for the prognosis of PC. There was also no overlap with our main reported results.

Our study has two relative advantages or differences. On the one hand, we utilized a wider range of transcriptome data sources and a larger data volume, which enhanced the reliability and universality of the research results. For example, we used a large number of bulk and single-cell transcriptome data. On the other hand, and most critically, as mentioned in the manuscript, we report 22 differentially expressed genes with prognostic value, all of which are not well understood in PC. In particular, the predictive value of *DCBLD2*, *OCIAD2*, and *SAMD9*, as well as their expression in single-cell sequencing and clinical samples, has been explored, which has supplemented and enriched this knowledge. These are aspects of our study that are different from other studies. Of course, it is inevitable that our current research also has many shortcomings. For example, you mentioned below that the current study on OCIAD2 has insufficient in-depth mechanistic research, and we mentioned in the manuscript that the prognostic value of proteins has not been studied in clinical cohorts, etc. In the future, we will strive to improve these studies.

2. More seriously, the Yin 2024 Gene paper (ref. 52), reported very similar discoveries on OCIAD2, although some differences in the proposed downstream mechanisms with the authors.

Response: Indeed, during the period of writing our article, it was found that there was a study reporting *OCIAD2* in pancreatic cancer. Nonetheless, our findings differ in many ways. For example, we have reported a number of other prognostic genes, and we have developed and evaluated prognostic models based on *OCIAD2* and other genes and pathological features. We also analyzed the expression of *OCIAD2* and others in single-cell sequencing data of normal pancreas, adjacent to cancer, primary cancer, and metastatic cancer. Yin et al. focused on the function of *OCIAD2* in pancreatic cancer cells and found that it was related to the AKT pathway. In fact, our studies complement each other. In addition, we also found many other different pathways based on transcriptome, such as JAK-STAT and cell cycle pathways. Certainly, changes in the AKT pathway were also observed in our study (Figure 5F).

3. Even for the mechanisms, the data are very weak. It is unclear how OCIAD2 regulates the hypothesized JAK-STAT pathway in PCa cell lines or specimens.

Response: We agree with your suggestion that further in-depth mechanistic studies are necessary. However, due to the limited research conditions, we can only carry out these studies at the transcriptional level at present. Encouragingly, transcriptome data based on both cell lines and clinical samples showed a significant correlation between *OCIAD2* expression and key molecules in the JAK-STAT pathway, such as STAT1. Therefore, we will conduct more in-depth research when conditions permit in the future.

4. The therapeutic value of OCIAD2 is also limited. No specific drugs for patients with OCIAD2 overexpression or silencing were validated.

Response: Our previous drug susceptibility analysis found a positive correlation between *OCIAD2* expression and the IC50 of gemcitabine (Figure 7A). According to your request, we conducted a preliminary drug sensitivity analysis in the BxPC-3 and SW1990 cell lines, and the results showed that with an increase in drug concentration, the inhibition rate of gemcitabine on the *OCIAD2* knockdown group increased significantly (as shown below). In the future, we will undertake further work in this area based on your suggestions. Thank you for your suggestion.

Inhibition rates of *OCIAD2* knockdown and control groups with different drug concentrations of gemcitabine in BxPC-3 and SW1990 cell lines.

**Reviewer 3**

The authors used OCIAD2 as a novel prognostic and therapeutic biomarker for pancreatic cancer and conducted a study based on transcriptomic signature and bioinformatics analysis. This work is interesting, and the paper need to be revised as follows:

1. To strengthen the findings, the authors should also consider Friedman’s test and Shapiro–Wilk test statistical tests.

Response: Dear professor, thank you for your valuable suggestions. According to your requirements, we have found that the Shapiro-Wilk test is used to evaluate whether the data follows a normal distribution, and Friedman's Test is used to determine whether multiple related samples (such as repeated measurements, compatibility group designs) come from the same distribution. There are not many such experimental designs involved in this manuscript of ours. Therefore, we thought you might want us to perform a further analysis of the prognostic model in Figure 2C to compare its ability to predict survival at 1, 3, and 5 years in different data sets. Therefore, we added the C-index based on the model to predict the prognosis of pancreatic cancer patients with ICGC (ICGC-PC-AU: 1-year: 0.6, 3-year: 0.75, 5-year: 0.85). The results showed that the C-index of each period in each data set followed the normal distribution (below figure, based on the Shapiro-Wilk test), and the Friedman's test results showed that there was no significant difference in the performance of the prognostic model in predicting the survival of patients in each data set at 1,3, and 5 years. The other main analyses, such as differentially expressed genes and survival analysis, have their own specific statistical analysis and testing methods, so we did not use the above two methods for statistical testing.

2. Provide a confusion matrix of the proposed model atleast. This would help illustrate the distribution of true positives, false positives, true negatives, and false negatives, in a clearer view of model performance across different classes.

Response: According to your request, we have added the prognostic model 1,3, and the 5-year confusion matrix to Supplement 3.

Supplement 3. Confusion matrices of prognostic models 1, 3, and 5 years.

3. The experimental results could further be clearly presented, either as an additional column in the table or in a separate statistical comparison section.

Response: Dear Professor, in this study, the transcriptome sequencing data performed after OCIAD2 knockdown in the BxPC-3 cell line belong to the data generated by our experiments. We have archived the raw data to the National Center for Bioinformation (GSA-Human: HRA007928), and we provide row count and FPKM data in Supplement 6.1. Now, according to your request, we have added the differentially expressed genes and related statistical data based on this as Supplementary 6.2-3. Because our subsequent main analysis is conducted around these differentially expressed genes, we hope these meet your requirements.
