# Supplementary material for: OCIAD2 as a novel prognostic and therapeutic biomarker for pancreatic cancer: A study based on transcriptomic signature and bioinformatics analysis
Source: PLoS Comput Biol. 2025 Oct 7;21(10):e1013566. doi: 10.1371/journal.pcbi.1013566 (PMC12517509; doi:10.1371/journal.pcbi.1013566)
Supplement: Attachment — Submitted filename: Response to Reviewers-2.docx [file pcbi.1013566.s010.docx]

Dear Editor and Reviewer,

On behalf of my co-authors, we thank you very much for allowing us to revise our manuscript. We also appreciate the reviewers very much for their positive and constructive comments and suggestions on our manuscript entitled “OCIAD2 as a novel prognostic and therapeutic biomarker for pancreatic cancer: a study based on transcriptomic signature and bioinformatics analysis” (Manuscript Number: PCOMPBIOL-D-25-01246R1).

In a previous version, we revised the manuscript based on these comments and suggestions. Now, according to the suggestion of Reviewer #2, we verified the activation of STAT1/STAT3 after knocking down *OCIAD2* in two pancreatic cancer cell lines (BxPC3, SW1990). We responded to Reviewer #2 point by point according to the experimental results.

Once again, thank you very much for your comments and suggestions. And we hope that the revised manuscript can be accepted by PLOS Computational Biology. Thank you and best regards.

**Reviewers' comments:**

**Reviewer 2**

As I have mentioned in the previous comments, the current study needs to demonstrate its novelty against a number of similar publications. The mechanism part, which could be a good direction, remains weak in this revised version. Actually, it has been reported that OCIAD2 directly interacts with and activates STAT3 (https://pmc.ncbi.nlm.nih.gov/articles/PMC5943604/). I suggest the authors to verify whether this mechanism is the one in PAAD as well.

Response: Thank you for your suggestion. According to your suggestion, we detected the activation of STAT3 after knocking down the expression of *OCIAD2* in the BxPC3 and SW1990 cell lines. The results showed that the total protein and phosphorylated protein levels of STAT3 did not change significantly after *OCIAD2* was knocked down in these two pancreatic cancer cell lines, but the total protein and phosphorylated protein levels of STAT1 showed a downward trend, which was consistent with our previous analysis results (Figure 6), as shown below. Although our present results suggest that *OCIAD2* is associated with STAT1 but not STAT3 activation, it should be emphasized that these are preliminary results based on two cell lines. Further studies are needed in other cell lines and pancreatic cancer patient samples.

The activation status of STAT1 and STAT3 after knocking down *OCIAD2* expression. (A) Results in the *OCIAD2* knockdown and negative control groups of the BxPC3 cell line. (B) Results in the *OCIAD2* knockdown and negative control groups of the SW1990 cell line.
